# Citrus flush shoot ontogeny modulates biotic potential of *Diaphorina citri*

**DOI:** 10.1371/journal.pone.0190563

**Published:** 2018-01-05

**Authors:** Juan Camilo Cifuentes-Arenas, António de Goes, Marcelo Pedreira de Miranda, George Andrew Charles Beattie, Silvio Aparecido Lopes

**Affiliations:** 1 Departamento de Fitossanidade, Faculdade de Ciências Agrárias e Veterinárias, Universidade Estadual Paulista ‘Júlio de Mesquita Filho’, Jaboticabal, São Paulo, Brazil; 2 Departamento Científico, Fundecitrus, Araraquara, São Paulo, Brazil; 3 School of Science and Health, Western Sydney University, Penrith NSW, Australia; Public Library of Science, UNITED KINGDOM

## Abstract

The biology and behaviour of the psyllid *Diaphorina citri* Kuwayama (Hemiptera: Sternorrhyncha: Liviidae), the major insect vector of bacteria associated with huanglongbing, have been extensively studied with respect to host preferences, thermal requirements, and responses to visual and chemical volatile stimuli. However, development of the psyllid in relation to the ontogeny of immature citrus flush growth has not been clearly defined or illustrated. Such information is important for determining the timing and frequency of measures used to minimize populations of the psyllid in orchards and spread of HLB. Our objective was to study how flush ontogeny influences the biotic potential of the psyllid. We divided citrus flush growth into six stages within four developmental phases: emergence (V1), development (V2 and V3), maturation (V4 and V5), and dormancy (V6). *Diaphorina citri* oviposition and nymph development were assessed on all flush stages in a temperature controlled room, and in a screen-house in which ambient temperatures varied. Our results show that biotic potential of *Diaphorina citri* is not a matter of the size or the age of the flushes (days after budbreak), but the developmental stage within its ontogeny. Females laid eggs on flush V1 to V5 only, with the time needed to commence oviposition increasing with the increasing in flush age. Stages V1, V2 and V3 were most suitable for oviposition, nymph survival and development, and adult emergence, which showed evidence of protandry. Flush shoots at emerging and developmental phases should be the focus of any chemical or biological control strategy to reduce the biotic potential of *D*. *citri*, to protect citrus tree from Liberibacter infection and to minimize HLB dissemination.

## Introduction

The Asiatic citrus psyllid *Diaphorina citri* Kuwayama (Hemiptera: Sternorrhyncha: Liviidae) is the only known vector of the fastidious phloem restricted α-Proteobacteria, '*Candidatus* Liberibacter asiaticus' and '*Ca*. L. americanus', associated in Brazil with huanglongbing (HLB), the most devastating disease of citrus [1,2]. It was first recorded in Brazil in 1942 [3] and subsequently spread throughout the main citrus producing regions of the country [4] but did not become economically important until after the first report of HLB in citrus orchards in São Paulo State (SPS) in 2004 [5]. Planting of certified nursery citrus trees produced in insect-proof screen-houses, elimination of symptomatic trees to reduce inoculum sources, monitoring and insecticide applications [6–8], were subsequently implemented in order to minimize the spread of the disease. Insecticide applications during the citrus dormant seasons (late autumn and winter), when adult insects survive by feeding on mature leaves, have also been recommended [9–13]. Despite these efforts the disease spread and 46.2 million trees had been removed by 2016 [14]. Recent estimates based on observations of symptoms in the field indicate that further 16.73% of the 191.7 million productive trees, most in SPS, are currently infected [15].

The difficulty in successfully managing HLB is related in part to the criteria used to determine the most appropriate timing and frequency of insecticide applications. In SPS these criteria are based mainly on the presence of adult psyllids captured on yellow sticky traps (YST). However, the number of adults per YST may not reflect the extent of psyllid populations [16], as they are influenced by trap density and location within orchards, positioning within tree canopies, population densities of the psyllid, weather conditions, and the nature and abundance of new flush growth [16–22]. Moreover, the impact of flush growth ontogeny on populations of *D*. *citri* is not clearly understood.

In SPS, which has a temperate/subtropical climate, citrus trees usually produce new growth twice annually in relatively well-defined cycles, one related to plant growth in summer-autumn, and one related to flowering and fruiting in spring. The flushes show sympodial growth as they pass through predicable and recognizable stages of development (termed ontogeny) [23] that are genetically and environmentally governed, with temperature, photoperiod, solar radiation and rainfall, playing important roles [24–30].

Although eggs, nymphs or adults of *D*. *citri* can be found on reproductive flushes, vegetative flushes are apparently the main sites for psyllid development. This explains the strong association between vegetative flush phenology and the dynamics of *D*. *citri* populations in citrus groves [31–34], and the rate of spread of HLB [35]. The psyllid is attracted to flushes that offer optimal conditions for feeding and oviposition, especially those at the very initial stage of development, by a combination of chemical volatiles and visual stimuli [36–41]. It has been known since Husain and Nath [42] published the first major study on *D*. *citri* in 1927 that oviposition occurs almost exclusively on very young flushes. They wrote “The period of greatest activity of the insects and most rapid increase in their numbers corresponds with that of the sprouting of new shoots and the appearance of new leaves” [42]. Other authors have reported that females lay their eggs in the growing tips of young host plants, preferring flush growth < 6 mm in length [43–46] and that most eggs are laid within 14 days of new growth commencing [43]. Initiation of oogenesis, and subsequent maturation of eggs within ovaries, is closely related to the presence of buds [47–49]. ‘Feather flush’ has been cited as the growth stage on which eggs are laid [33,50–52] but this term, incorrectly ascribed to Chavan and Summanwar (1993) [34] by Halbert and Manjunath (2004) [50], appears to be the first of five stages related to monitoring of the black citrus aphid *Toxoptera citricida* (Kirkaldy) (Hemiptera: Sternorrhyncha: Aphididae) [53] and a stage of growth with ‘leaves still folded’.

Although an overall description of flush development is available in the literature [54], no detailed phenotypical illustration of each stage during shoot ontogeny exist, as well as the risk that each growth stage of flushes represents to the development and increasing of *D*. *citri* population. The goal of this study was to (i) define, clearly describe and illustrate the stages during flush growth ontogeny on citrus, and (ii) determine the relative importance of each stage on insect oviposition, nymph survival and adult emergence.

## Material and methods

The experiment was carried out twice at the Plant Protection Department of the São Paulo State University, in Jaboticabal, SPS, Brazil (21.2522° S, 48.3257° W), in two different environments: (i) a 3 × 3 × 3 m temperature-controlled environmental room (CER) (average 26 ± 2°C, 70 ± 5% RH, 12 h photoperiod) with artificial light provided by 16 fluorescent bulbs (32 W each) and 5 incandescent lamps (250 W each) yielding 3500 to 4000 lux, and (ii) a 8 × 12 × 5 m screen-house (SH) under ambient temperatures (average 25°C, max. 42°C, min. 12°C) and relative humidity (average 53%, max. 93%, min. 15%). Data on temperature and relative humidity were recorded with a HT-500 data logger (Instrutherm, SP). The experiments were carried out from March to April 2014 (autumn) inside CER, and from September to October 2014 (spring) inside SH.

### Plant material

Two-year-old nursery trees of 'Valencia' sweet orange (*Citrus sinensis* (L.) Osbeck.) grafted on 'Swingle' citrumelo (*Citrus paradisi* MacFaden × *Poncirus trifoliata* (L.) Raf.–based on [55]) were maintained in 4.7 L plastic pots containing substrate composed of 80% *Pinus* sp. bark, 15% vermiculite and 5% charcoal (Multiplant citrus®; Terra do Paraiso, Holambra, SP). Before starting the experiments, the plants were kept in greenhouse irrigated four times a week and ferti-irrigated biweekly with 100 mL pot^-1^ of a nutritive solution containing Ca(NO_3_)_2_, MAP, MgSO_4_, Cu, Zn, Mn-EDTA, (NH_4_)_2_MoO_4_, and Fe-EDTA, at concentrations of 1.35, 0.111, 0.4, 0.015, 0.01, 0.0075, 0.00045, 0.075 g L^-1^, respectively.

### Insect rearing

Fifteen to twenty *D*. *citri* adults of each sex from a colony on healthy orange jasmine plants (*Murraya exotica* L.) at Fundecitrus (Araraquara, SP) were confined for oviposition in sleeve cages for 5 days in the CER, on immature growth flushes of previously uninfested orange jasmine plants, and then removed to allow eggs laid to hatch and nymphs to develop to a new generation of adults. After eclosion the adults were separated daily and placed individually in 50 mL glass test tubes for sexing [56]. The insects were then transferred to new non-infested orange jasmine plants, grouping by sex. Daily insect separation of emerging adults ensured collection of unmated adults [57]. A manual aspirator, made of a mesh-covered plastic Pasteur pipette inserted into a plastic tube was used to collect the adults, and a 30× magnification hand lens was used to distinguish males from females. Given that adult emergence fits Gaussian curve, the few adults that emerged during the first three days were discarded. Only those at the top of the bell shape of the emergence growth curve (days 4 to 7) were used. These procedures allowed the use of a relatively uniform group of insects that were further maintained on orange jasmine in the CER for additional 15 days before use in the experiments. This methodology was adapted from Skelley & Hoy [58].

### Ontogenesis of flush shoots

**Fig 1:**
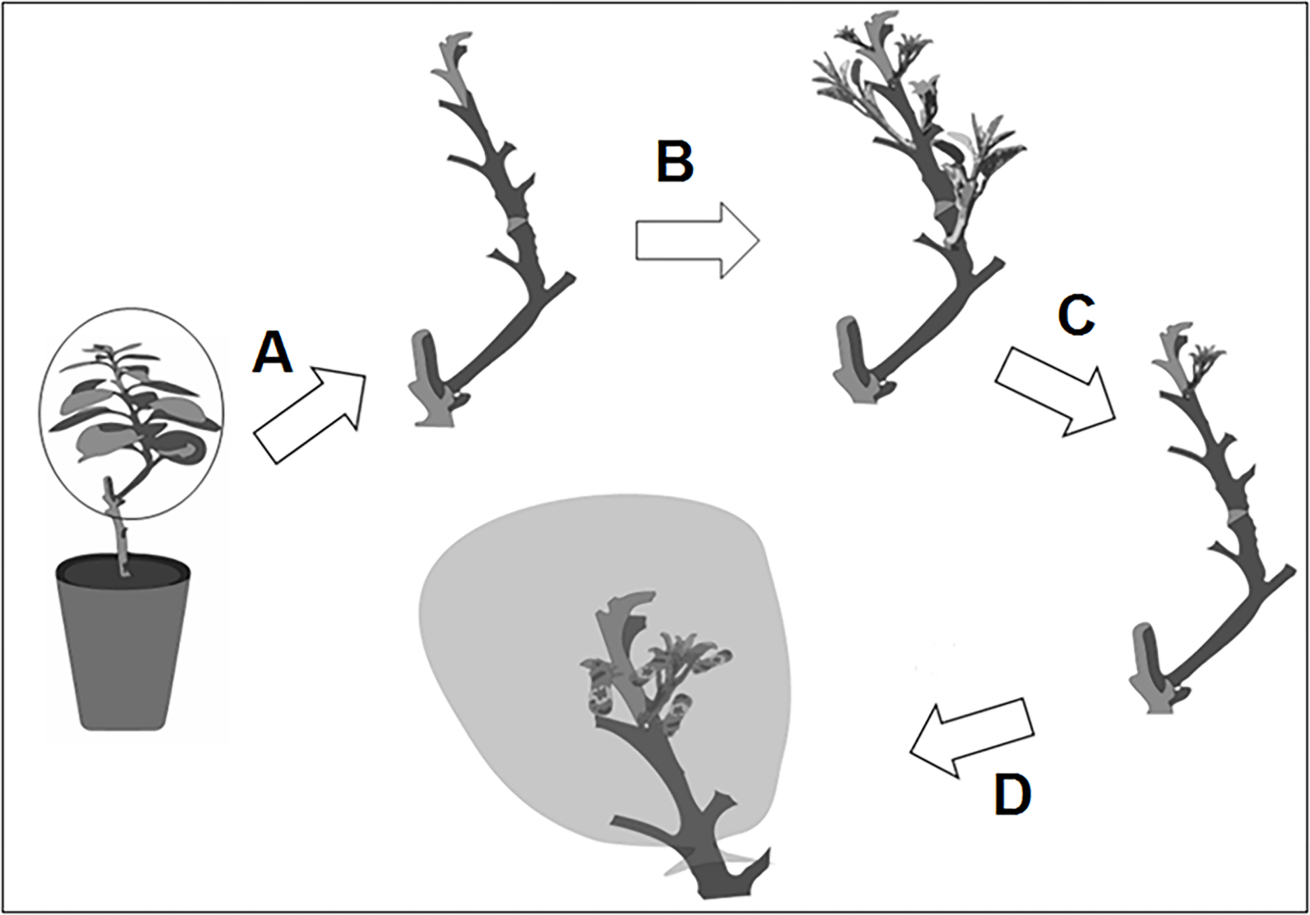
General schematic representation of treatments. (A) Pruning and defoliation. (B) New flush shoots. (C) Selection of flush shoot at the beginning of stage V2. (D) Pruning and selection period of the remaining groups for final standardisation and insect caging.

Previous observations of potted plants in greenhouses and mature trees in orchards led us to divide the growth of citrus flush shoots into six discrete stages of development. To describe the stages and their durations, four healthy two-year-old potted plants of ‘Valência’ on ‘Swingle’ citrumelo rootstocks were pruned 20 cm above the bud union and fully defoliated. When the 3^rd^ or 4^th^ bud below the pruning site started swelling, its length was measured with a Vernier calliper (Mitutoyo 530 Series, Suzano, SP, Brazil). Any other subsequent new shoot was detached (Fig 1A–1C).

### Influence of flush ontogeny on the biotic potential of *Diaphorina citri*

To evaluate the influence of flush ontogeny on *D*. *citri* multiplication, 100 potted ‘Valencia’ plants were divided into five groups, each comprising 20 plants. The plants were then sequentially pruned and defoliated weekly over a 4-week interval (Fig 1A to 1C). Using a sleeve cage (Fig 1D), two unmated 15-day-old *D*. *citri* couples were confined for mating and oviposition on each flush stage. In the CER the insects were confined for 72 h. In the SH, the flushes were observed for the presence of eggs every 24 h and, when present, the adults remained confined for additional 48 h to allow 72 h for oviposition. Eggs and nymphs were then counted, and emerging adults collected every four days (CER) or daily (SH). All adults were stored at -20°C in 5 × 20 cm transparent plastic bags for further sex identification using a stereo-microscope. Egg, nymph, and total viability (V_e_ = [nymph/eggs]*100, V_n_ = [adults/nymphs] *100, and V_t_ = [adults/eggs] *100, respectively), as well as sex ratio (SR = % female) and duration of egg-to-adult cycle, were determined for both ambient, whereas time to oviposition and synchrony of male/female emergence were determined only for SH. Egg-to-adult cycle was estimated for each flush shoot (replicate), summarizing the period from the second oviposition day (24 to 48 h) until previous day when first adult appeared, plus the numbers of days between first adult appeared until 50% of adults emerged, this one estimated by a nonlinear regression.

### Statistical analysis

To describe flush shoot elongation, several non-linear regression analyses were carried out. The final model was chosen based on the Akaike Information Criteria (AIC) [59], for which the simplest and smaller model that maximizes the goodness-of-fit is the best for explaining the data [60]. Prior to statistical analysis, data on number of eggs and percentages were log (*y’ = Log*_*10*_ *(y + 1)*) and *arcsine* (y’ = arcsine(sqrt(y/100))*180/π) transformed, respectively [61]. The effect of the different flush stages on psyllid biology was evaluated by analysis of variance using the general linear model (GLM) procedure. Each ambient was analysed separately. When significant differences were detected, the means were compared by Tukey-HSD test. Sex ratio and oviposition percentages were analysed by the Chi-square test, or by Fisher’s Exact test when the frequencies were less than 5 [61]. To evaluate the distribution of adult emergence patterns, two procedures were applied: (i) a linear regression of the quantiles (1, 5, 10, 25, 50, 75, 90, 95, 99) (for the female emergence as a function of male emergence) [57], and (ii) a Linear Trend test complemented with analysis of the adjusted residual [62]. Interpretation of adjusted residuals was made according to Haberman [63], for which absolute values greater than │1.96│ (*P* = 0.05) or │1.65│ (*P* = 0.10) represent lack of independence, or an indication that the observed frequency (*X*_*ij*_) is more variable than should be. The Statgraphics Centurion XVII software (Statpoint Technologies Inc.) was used, with *P* < 0.05 for all analyses.

## Results

### Flush shoot ontogenesis

**Fig 2:**
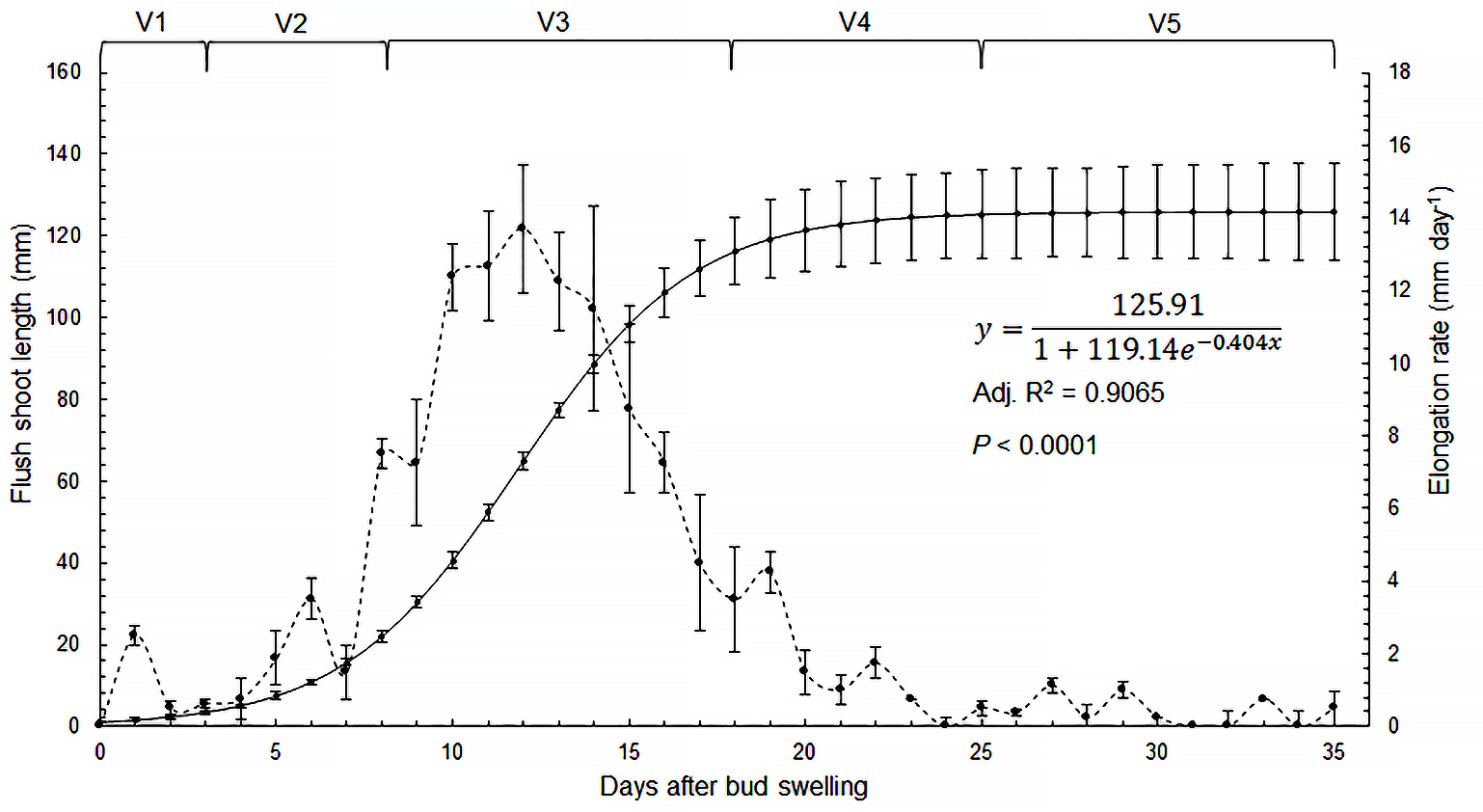
Flush shoot growth. Adjusted curve describing flush shoot length (continuous line; sigmoidal logistic curve selected based on the AIC [59]), elongation rate per day (dashed line) and approximate duration and length of each stage during ontogeny (vertical lines are standard error of the mean of the original data, n = 4 plants) (See S1 Apendix).

**Fig 3:**
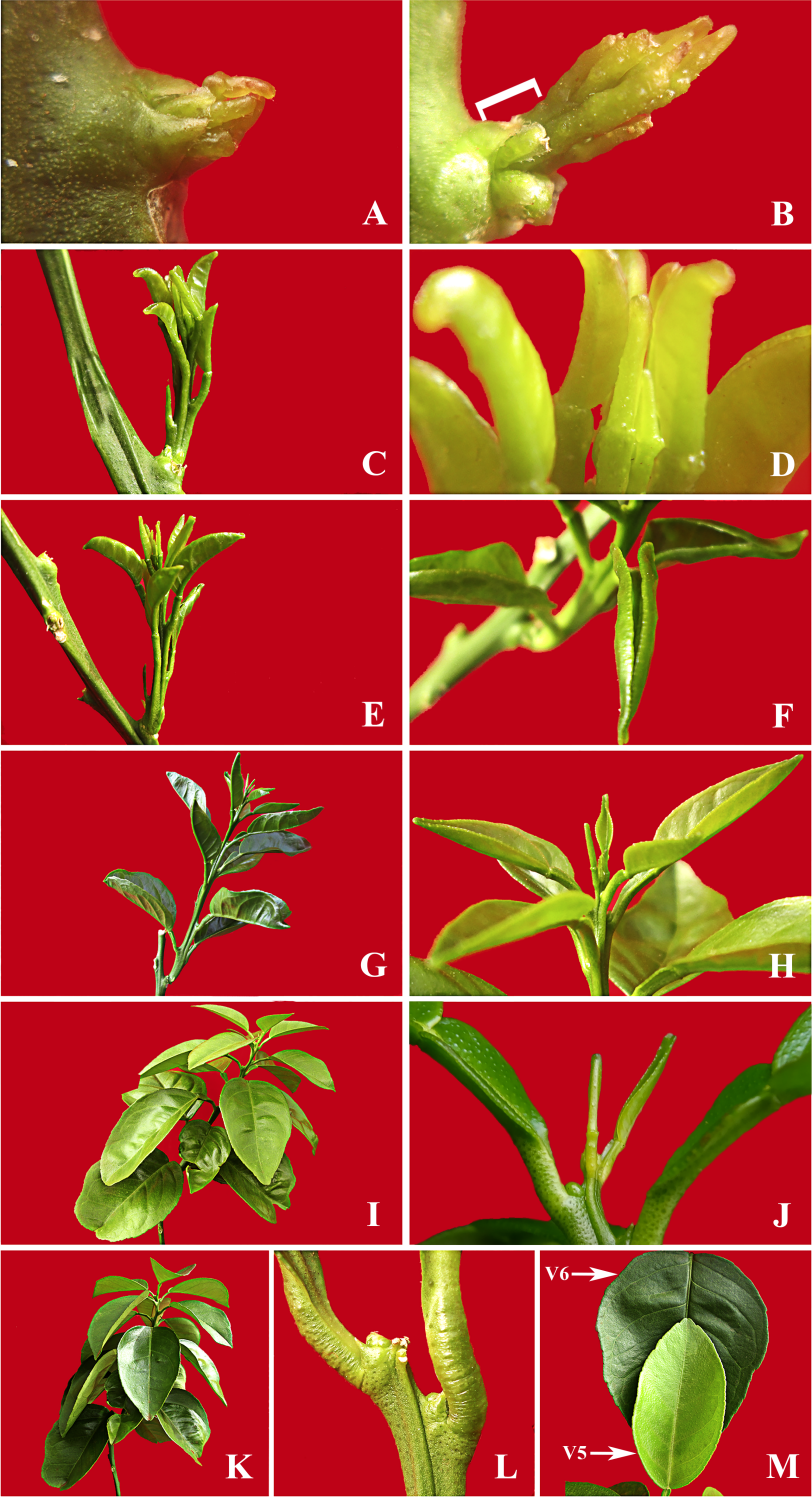
Ontogeny of flush shoot of ‘Valencia’ sweet orange plants. Stage V1: from bud swelling (A) to bud emergence with less than 2 mm flush stem (white square bracket) (B). Stage V2: from initial stem elongation with separation between basal petioles (C) but all margins of the leaves remaining closed (D). Stage V3: from initial leaf blade expansion and separation of the flush axis (E) with margins of lower leaves opening (F) until new leaves emission stops. Stage V4: from unfolding of all leaf (G) and final leaf number defined (H) to shoot tip chlorosis. Stage V5: leaves fully expanded, green-light yellow coloured, gradual hardening from top to base (I), and shoot tip chlorosis and/or abscission (J). Stage V6: vegetative flush completely matured with leaves fully hardened and green-dark (K) and with dormant buds (L). Comparison between fully expanded, partially hardened and green-light-yellow leaf from a V5 shoot and one fully expanded, hardened and final green-dark leaf from a V6 flush (M). For relative sizes and duration of each stage see Fig 2.

Flush shoot elongation in the SH fitted a sigmoidal logistic curve (Fig 2) spanning four developmental phases over an average 35 days. **Phase 1, emergence,** that comprised a single stage, **V1** (Vegetative 1), that commenced with bud swelling and opening of the protective scales (Fig 3A and 3B). In this phase, elongation rates reached a maximum of ≈ 2.5 mm day^-1^ over an interval of 3 days (Fig 2). **Phase 2,** developmental, comprised two stages, **V2** and **V3**. At the beginning of this phase (V2), which lasted about 5 days, flush elongation rates were ≈ 1.5 to 3 mm day^-1^ (Fig 2). There was an initial expansion of the lamina of the basal leaves but the margins stayed folded inwards so that the adaxial leaf surfaces were not visible (Fig 3C–3D). Latter in V3, which lasted about 9 days, the flush elongation was ≈ 10 mm day^-1^, reaching a maximum of ≈ 14 mm day^-1^ at the middle of the stage, followed by a decline to ≈ 5 mm day^-1^ (Fig 2). During this stage the leaf margins opened and adaxial leaf surfaces became visible (Fig 3E to 3F). **Phase 3,** maturation, comprised two stages, **V4** and **V5**. Flush shoot elongation continued but decreased very quickly from ≈ 5 mm day^-1^ at the beginning of **V4**, which lasted about 7 days, to less than 0.25 mm day^-1^ during **V5**, which lasted about 11 days (Fig 2). **V4** began when emission of new leaves stopped and was the period when the leaves gradually hardened from the top to the base with the colour changing from bright green to opaque light green-yellow (Fig 3G to 3H). During **V5,** which began when a shoot tip chlorosis and posterior abscission was evident, the leaves became almost fully expanded and hardened (Fig 3I to 3J), changing from light green-yellow to dark green. **Phase 6,** dormancy, comprised one stage, **V6**, characterized by fully expanded mature dark-green leaves (Fig 3K–3M).

### Influence of flush shoot ontogenesis on biotic potential of *D*. *citri*

#### Controlled environmental room

**Fig 4:**
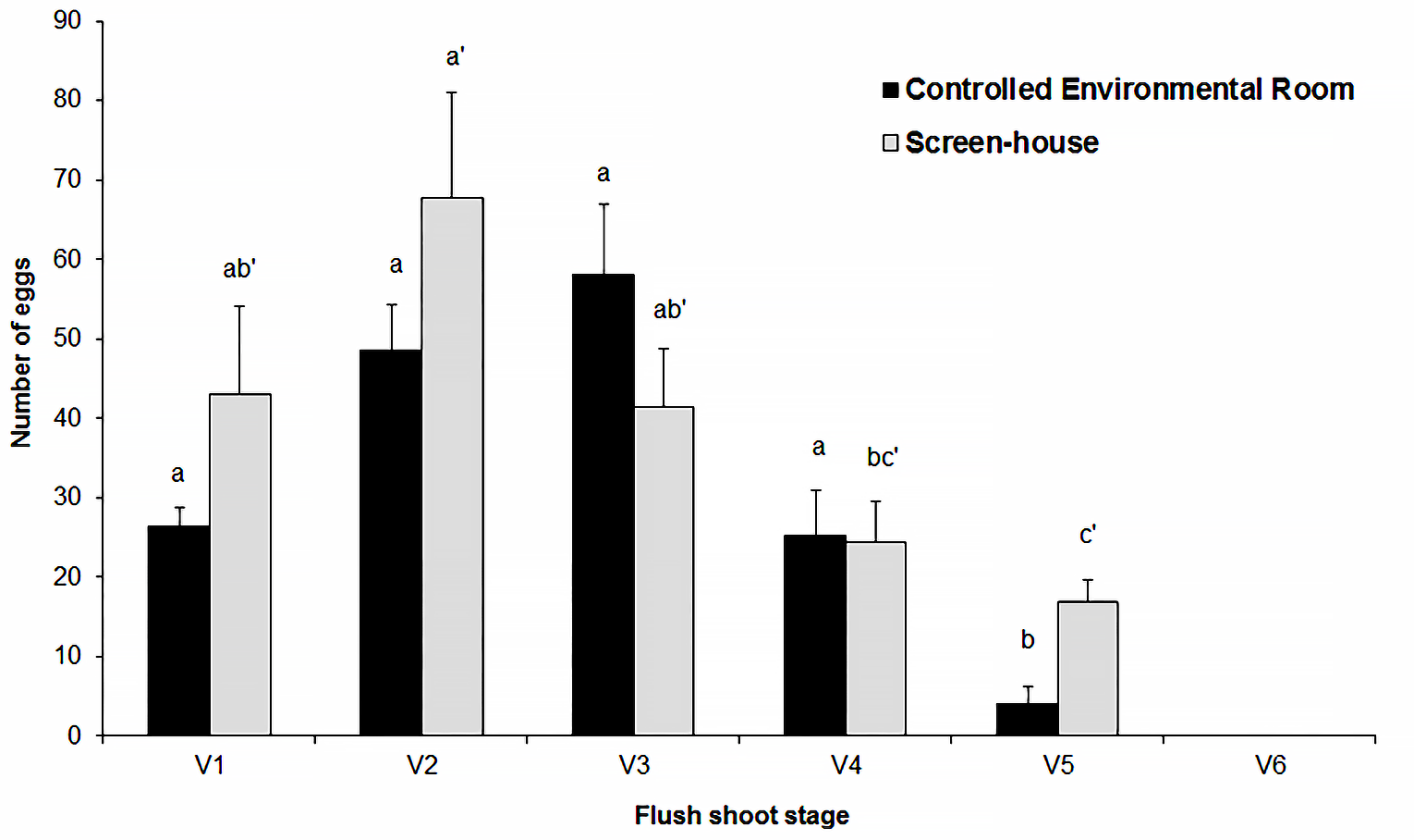
Oviposition by *Diaphorina citri*. Means (±SEM) of the number of eggs laid by *Diaphorina citri* on different flush stages of ‘Valencia’ sweet orange plants grown under controlled environmental room (black bars; 26°C ± 2, 70% RH ± 5, 12h photoperiod) and screen-house (grey bars; 25°C ±8, 53% ±21 HR and 12h photoperiod) conditions. No eggs were laid in stage V6 in both ambient. Bars with different letters differ by Tukey-HSD test, *P* < 0.05 (lowercase for CER and lowercase with quote for SH; no comparisons were made between ambient).

The females were allowed to lay eggs for 72 h. Oviposition frequency was dependent on the growth stage of the flush (Fisher’s Exact test = 21.872, P = 0.0003). Eggs were detected on 100, 94.7, 87.5, 85.7 and 44.4% of the plants with shoots at stage V1, V2, V3, V4, and V5, respectively. Eggs were not detected on V6 shoots. Flush stage also influenced oviposition (*F* = 16.7; df = 4; *P* < 0.0001), with highest percentages of eggs per shoot recorded on V2 and V3 (29.92% and 35.73%) flushes in contrast to V1 (16.29%), V4 (5.53%) and V5 (2.53%) (n ≈ 162; Fig 4) (See S2 Appendix).

While flush stage did not affect egg viability (*F* = 1.13; df = 4; *P* = 0.3532), it strongly affected nymph survival (*F* = 164.45; df = 4; *P* < 0.0001) with highest percentages of live insects found on V1 to V3 flushes. V5 flushes were unsuitable for development of nymphs. Nymphs that did hatch from eggs laid on these flushes died during the 1^st^ or 2^nd^ instar.

The negative impact of flush stage on nymph survival reflected on *D*. *citri* total viability, namely, the percentage of individuals that completed their life cycle (*F* = 96.13; df = 4; *P* < 0.0001). Emerging (V1) or developing (V2 and V3) flushes produced highest numbers of adults, with no significant differences among populations on life span (average 15 days; *F* = 1.06; df = 3; *P* < 0.3751) or final sex ratio (60% females; Chi-square = 6.523, df = 3, *P* = 0.0888, n = 1435). On average, 18.13, 34.57, 45.96, 1.34, and 0% of the adults emerged from flushes V1 to V5, respectively (See S2 Appendix).

#### Screen-house

**Fig 5:**
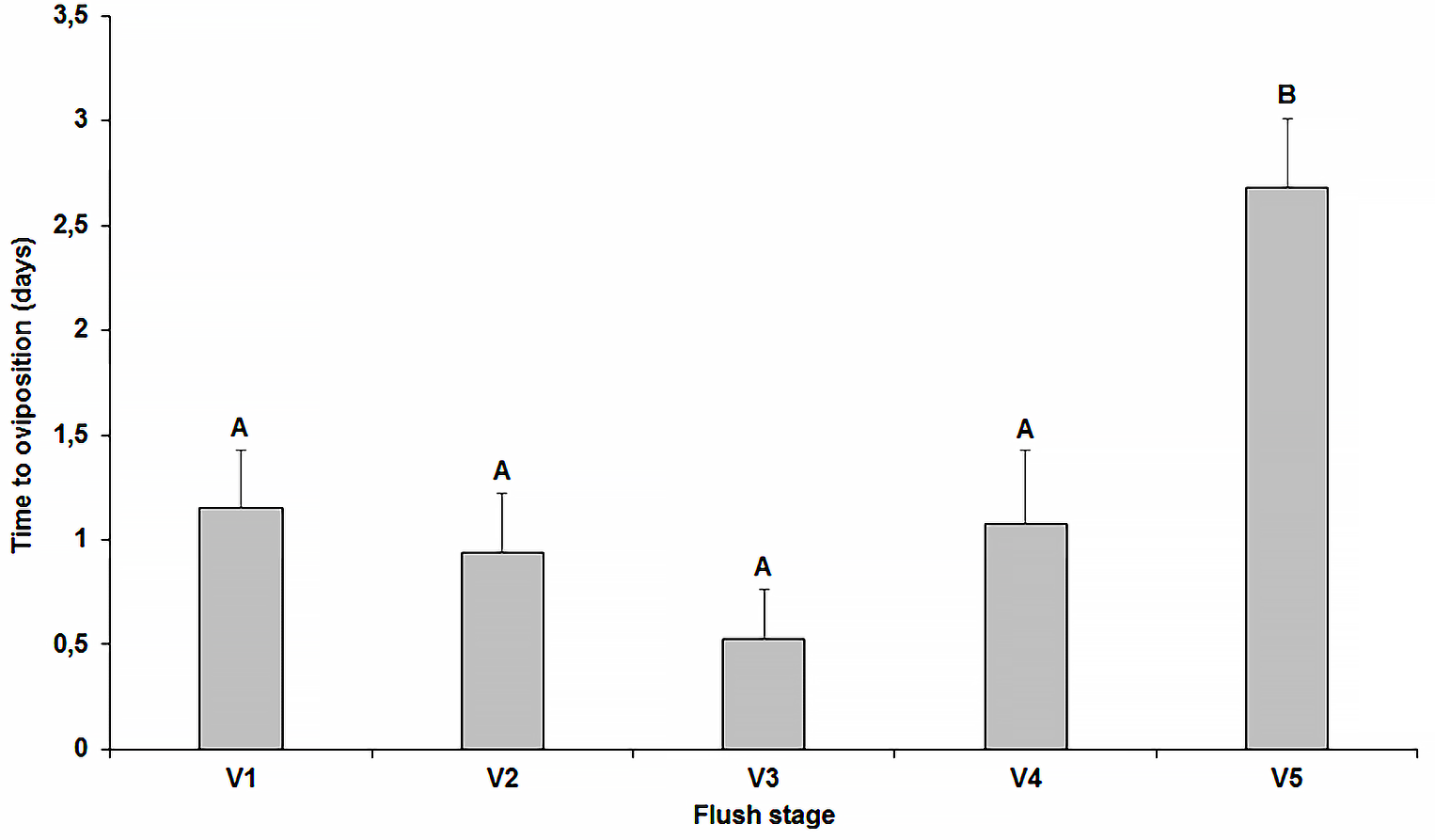
Time to begin oviposition. Mean (±SEM) values of time needed for females of *Diaphorina citri* to start oviposition on new shoots of citrus under screen-house conditions (bars with different letter differ statistically by Tukey-HSD test, *P* < 0.05).

Unlike the experiment conducted in the CER, females in SH were allowed to lay eggs for variable time periods with observations over 72 h commencing when the first eggs were observed on the flushes. The females laid eggs on most flushes (100% of V1 to V4 and on 93.3% of V5), but the time needed for commence oviposition was significantly influenced by flush stage (*F* = 9.18; df = 4; *P* < 0.0001), with longer intervals on V5 (≈ 3 days) than on V1 to V4 (≈ 1 day) (Fig 5). Flush stage also influenced egg number (*F* = 7.23; df = 4; *P* < 0.0001) (Fig 4) with the highest values recorded on V2 flushes in contrast to newly emerged V1, developing V3, and mature V4 and V5 flushes. On average, 22.24, 34.96, 21.36, 12.67 and 8.76% of the eggs (n ≈ 194) were laid on V1 to V5 flushes, respectively (Fig 4). (See S2 Appendix).

As observed under CER conditions, flush stage did not significantly influenced egg viability (*F* = 1.40; df = 4; *P* = 0.2448). Nonetheless, nymph viability on V1 to V3 flushes was significantly higher than on V4 or V6 (*F* = 20.54; df = 4; *P* < 0.0001). The percentage of individuals able to complete their life cycle was also significantly influenced by flush stage (*F* = 21.59; df = 4; *P* < 0.0001), with the developing flushes V2 and V3 being the most suitable, and V1 and V4 and V5 flushes the least suitable (Table 1). On average, 21.53, 44.39, 24.71, 6.61, and 2.76% of the total number of adults emerged from flushes V1, V2, V3, V4, and V5, respectively. Flush stage did no impact the duration of egg-to-adult cycle (average 23.3 days; *F* = 2.21; df = 4; *P* = 0.0759).

**Table 1:** Mean values (± SEM) of eggs, nymphs and total viability of *Diaphorina citri* on flush shoot stages of 'Valencia' sweet orange plants grafted on 'Swingle' citrumelo under controlled environmental room (CER) and screen-house (SH) condition.

Flush shoot stage^x^	n^y^		Viability (%)					
			Egg^z^		Nymph^z^		Total^z^	
	CER	SH	CER	SH	CER	SH	CER	SH
V1	9	13	83,9±1,7a	80,3±2,4a	79,1±4,0a	51,9±8,4b	66,5±3,7a	43,3±7,3b
V2	18	17	75,6±3,9a	84,6±3,4a	87,0±2,0a	79,4±4,5a	66,2±4,2a	68,2±5,5a
V3	14	19	75,7±3,3a	84,6±2,9a	85,5±2,7a	78,0±4,6a	64,6±3,5a	66,3±4,4a
V4	12	13	70,2±3,2a	81,4±2,1a	6,3±54,4b	32,8±6,7bc	4,9±3,5b	27,4±5,7bc
V5	8	28	73,9±6,9a	77,5±2,6a	0b	21,1±3,7c	0b	16,2±3,8c

^x^ Eggs were not laid on V6 flushes.

^**y**^ Number of replications.

^**z**^ Means with the same letter within the column did not differ statistically by Tukey-HSD test (P < 0.05) (comparisons were not made between both ambient).

**Fig 6:**
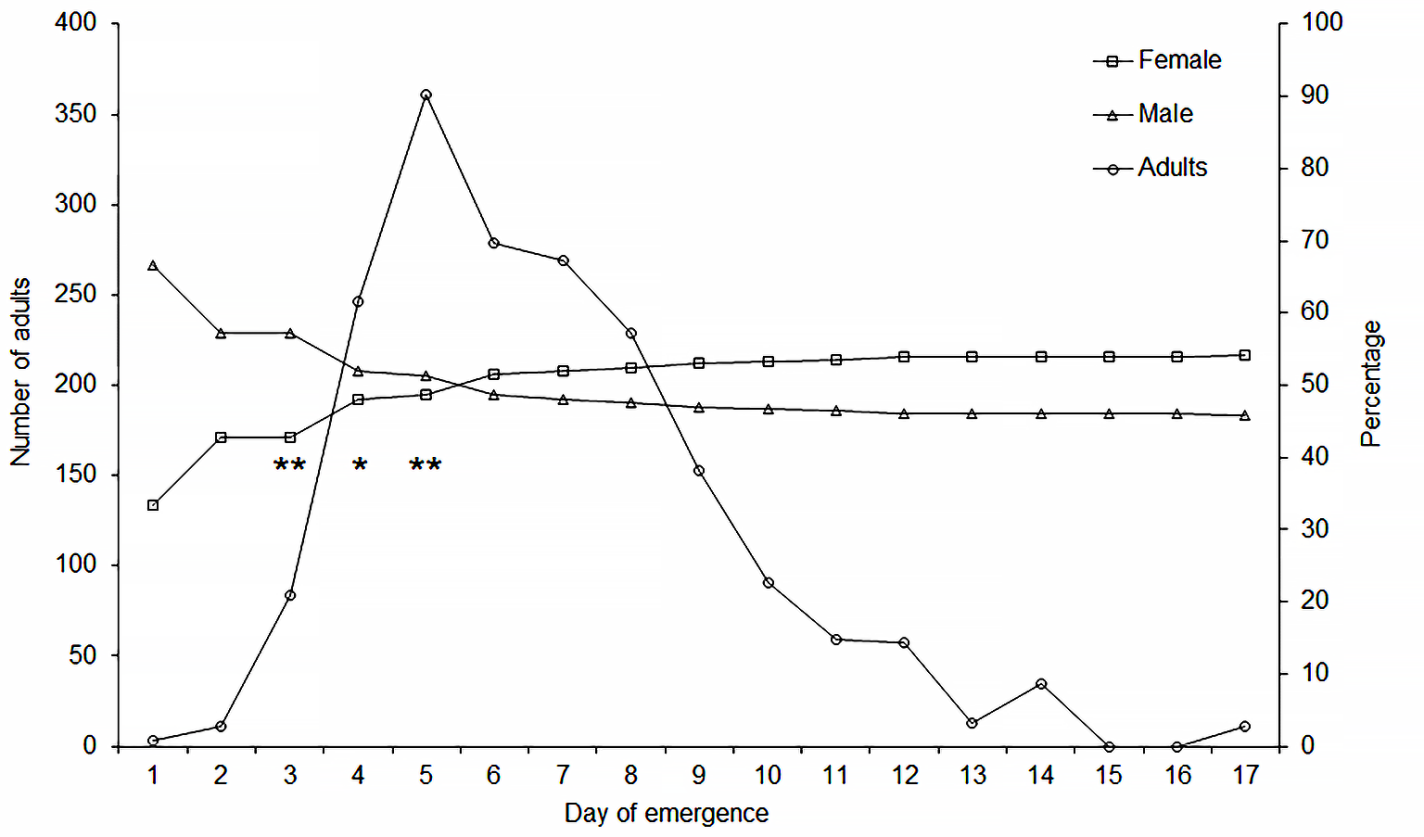
Schedule of *Diaphorina citri* emergence. Total number of adults emerged per day (circles) and synchrony proportion (%) of female (squares) and males (triangles) of *Diaphorina citri* in experiment performed under screen-house conditions (*, ** indicate adjusted residuals greater than │1.65│ for *P-value* < 0.10) and│1.96│ for *P* < 0.05, respectively).

In the SH insect removal and sexing were made daily. The proportion of male was higher in the first days but gradually declined until the fifth day, when the proportion of the cumulative number of emerged females surpassed that of male, reaching 54.13% on the 17^th^ day, the end of the emergence period (Fig 6). The analysis of the quantiles for female vs. male emergence revealed a positive correlation (F_1,7_ = 2236.64; *P* < 0.0001; r^2^ = 0.996; y = 1.181x – 15.862). A slope of 1.181 indicates a possible protandry, namely, emergence of males in higher proportion than females at the beginning of the emergence period (values below 1 would indicate protogyny). Furthermore, cumulative male/female emergence proportion was not independent from time (Linear trend test, *P* = 0.0012). Analysis of adjusted residuals for male at the 3^rd^ (2.05), 4^th^ (1.94) and 5^th^ (2.36) day of emergence, indicated that observed frequency of males was higher than expected, at exact probabilities of 0.0404, 0.0524, and 0.0183, respectively (See S3 Appendix).

In both ambient psyllid did not cause any apparent damage to stages older than V2, despite the large number of feeding individuals. However, the V1 flushes were smaller, contained fewer and atrophied leaves, and showed abscission of the feather new leaves primordia and shoot tip.

## Discussion

Our research related the maturity of flush growth on citrus and the development of *D*. *citri* from oviposition to adult emergence. In our first attempts to define the stages, we used the scales or criteria of Agustí et al. [64], Leong et al. [65], Stoller [66], and Yang et al. [37], which were developed based on flush size and colour, shoot elongation or leaf expansion. The simultaneous occurrence of all those variables limited their use. In our observations in greenhouses and orchards flushes of distinct sizes suggesting same phenotypic characteristics or level of tissue softness were frequently observed. This led us to develop a new scale which define six stages (V1-V6) of growth within four phases of flush development. It proved useful to distinguish the stages in our studies involving potted plants (S1 Fig) as well as in young and adult trees growing in the field (S2 Fig). We then determined the suitability of these stages for development of the psyllid.

*Diaphorina citri* females laid eggs on all growth flush stages with the exception of mature V6 flushes. Oviposition was higher on stages V2 and V3, and gradually declined as the shoots matured. Flush ontogeny also influenced the time required for females to commence oviposition, which took longer on harder tissues of stage V5 (≈3 days) than on the younger tissues of V1 to V4 (about 1 day). This suggests that changes in leaf hardness and its chemical composition [36,46,67] signal to females that the leaf tissues are not suitable for development of nymphs. Similar behaviour is exhibited by *Toxoptera citricida* [53], *Trioza erytreae* Del Guercio (Triozidae) [68], and citrus leafminer *Phyllocnistis citrella* (Lepidoptera: Gracilariidae) [69].

Flush ontogeny also impacted nymph viability, which was lower on stages V4 to V5. The few live young nymphs observed on older tissues moved quickly (which is in opposition to the suggestions in the way that younger nymphs walk less, apparently to reduce the risk of dehydration [70]), in contrast to those found on younger less lignified tissues. Since younger nymphs have shorter and weaker stylets [36], the relatively thicker leaf cuticles [71] and larger and deeper vascular vessels, characteristic of mature leaves [36,72], may have affected nymph probing and survival. Nutritional changes could be an additional factor. It is already known that the mineral composition of citrus leaves varies from organogenesis to maturation and senescence, with calcium remaining immobilized and nitrogen and potassium moving from older to younger tissues [73,74]. Nutritional variation in plant tissues may cause changes in tissue palatability and phloem sap quality, and consequently affect the activity of chewing and sucking insects [75–78]. For instance, changes in essential amino acid composition changed the performance of *Manduca sexta* Linnaeus [79], and the sucking insects *Bemisia tabaci* Gennadius [80,81] and aphids [38]. Changes in calcium supply also have shown to affect the integrity of plant cell wall and tissue hardness, leading to decreased damages caused by bacterial or fungal infections [82,83] and damages caused by feeding insects. Increased rigidity of leaf tissues in response to calcium supply caused attrition to the mouthparts of the chewing insects *Spodoptera exigua* Hübner, *Eldana saccharina* Walker, and *Deroceras reticulatum* Müller [84–86], and might impede *D*. *citri* stylet penetration.

The early emergence of males in proportion higher than that of females (Fig 6), suggests protandry in *D*. *citri*, a phenomenon that represents a reproductive advantage for insects with high fecundity rates [87], as is the case of *D*. *citri*. Protandry has been described in *Cardiaspina densitexta* Taylor [88] and *Cacopsylla pyri* L. (Psyllidae) [89] but not, in contrast to our observations, in *D*. *citri* [57,90]. It is possible that the relatively low numbers of adults observed in those previous studies with *D*. *citri* may have influenced the results and conclusions. The existence of protandry would benefit *D*. *citri* reproduction as males require more time to reach sexual maturity than females [57] and have shorter life spans [91,92].

Currently in SPS, effective measures to minimise the impact of HLB have been based on removal of symptomatic trees and area-wide suppression of *D*. *citri* populations [8]. Vector suppression has benefited from information generated through the “phytosanitary alert system”, which involves fortnightly inspection of some 18,000 YST distributed within the major citrus growing areas of the state [93]. The presence of new shoots on trees is also monitored. Regional and coordinated applications of insecticides start when numbers of adult psyllids per trap exceed set intervention thresholds (< 0.5 psyllid per YST) and when the density of new shoots indicates favourable conditions for rapid increases in psyllid populations. However, the shoot development criteria on which decisions are made are almost exclusively based on shoot size. Furthermore, psyllid records on YST would reflect a process that begun a few weeks before.

As shown in this study, shoot ontogeny provides a better evidence for phenotypic characterization and duration of the flush stages that mostly influence *D*. *citri* reproduction. Combined to an estimation of flush density using, for example, the methodology proposed by Hall and Albrigo [54], it may be possible to determine at a given moment the potential risk for psyllid multiplication in a particular orchard, and the best moment for psyllid control based on the proportion of most suitable flushes. Particular models that consider mainly the temperature for psyllid multiplication [94,95] and include the relative weight of each growth stage on psyllid biology. As shown in Fig 2, in the ambient conditions the study was conducted, the period of time most suitable to *D*. *citri* spanned around 20 days, from bud-break to the end of V3 flushes, when *D*. *citri* chemical control practices should be intensified. Outside that period, when leaf emission and expansion rate decrease quickly, it would be probably more reasonable to alternate insecticide or mineral oil applications with biological control [11,37,96–102]. Since a citrus orchard should be understood as a heterogeneous population of flush shoots, *D*. *citri* control also could be benefited from the establishment of a ‘reproduction’ or ‘potential risk’ threshold value, which could be estimated taking into consideration the risk each flush stage represents to *D*. *citri* reproduction, and the frequency of each stage in a given orchard at a given moment.

## Supporting information

S1 Appendix Raw data of flush shoot growth and development. (XLSX)

S2 Appendix Raw data of the performance of *Diaphorina citri* on different flush shoot stages. (XLSX)

S3 Appendix Raw data of the adult emergence. (XLSX)

S1 Fig Additional evidence of flush shoot growth stages from potted plants (‘Valencia’ sweet orange). (PDF)

S2 Fig Additional evidence of flush shoot growth stages from adult plants in the field (‘Valencia’ sweet orange). (PDF)
