# Supplementary material for: Citrus flush shoot ontogeny modulates biotic potential of Diaphorina citri
Source: PLoS One. 2018 Jan 5;13(1):e0190563. doi: 10.1371/journal.pone.0190563 (PMC5755881; doi:10.1371/journal.pone.0190563)
Supplement: S1 Fig — (PDF) [file pone.0190563.s004.pdf]

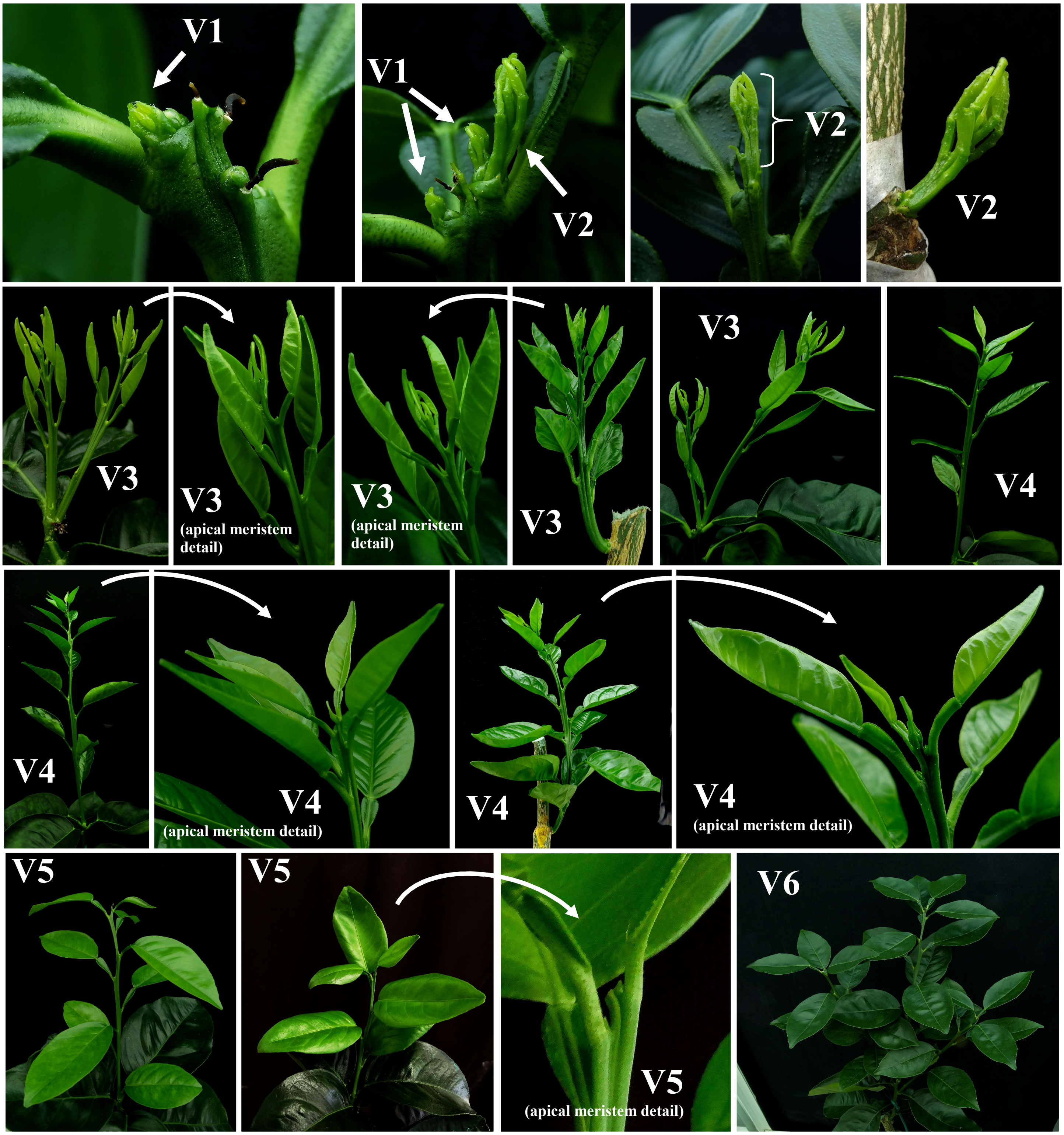

S1 Fig. Additional evidence of flush shoot growth stages from potted plants ('Valencia' sweet orange). Flush shoot developmental stage is not a question of size, but of phenotypical characteristics that indicate the onset of a certain developmental phenomena. Some shoots may have very few leaves (1 - 5), which makes the transition between stages faster.
